# Thermoresponsive and Reducible Hyperbranched Polymers Synthesized by RAFT Polymerisation

**DOI:** 10.3390/polym9090443

**Published:** 2017-09-13

**Authors:** Anna Tochwin, Alaa El-Betany, Hongyun Tai, Kai Yu Chan, Chester Blackburn, Wenxin Wang

**Affiliations:** 1 School of Chemistry, Bangor University, Deiniol Road, Bangor, Gwynedd LL57 2UW, UK; annatoffi.at@gmail.com (A.T.); alaaelbetany@gmail.com (A.E.-B.); chu037@bangor.ac.uk (K.Y.C.); chu230@bangor.ac.uk (C.B.); 2 Charles Institute of Dermatology, University College Dublin, Dublin 4, Ireland; wenxin.wang@ucd.ie

**Keywords:** hyperbranched polymers, thermoresponsive, reducible, RAFT polymerisation, disulfide diacrylate, hydrogels

## Abstract

Here, we report the synthesis of new thermoresponsive hyperbranched polymers (HBPs) via one-pot reversible addition-fragmentation chain transfer (RAFT) copolymerisation of poly(ethylene glycol)methyl ether methacrylate (PEGMEMA, *M*_n_ = 475 g/mol), poly(propylene glycol)methacrylate (PPGMA, *M*_n_ = 375 g/mol), and disulfide diacrylate (DSDA) using 2-cyanoprop-2-yl dithiobenzoate as a RAFT agent. DSDA was used as the branching agent and to afford the HBPs with reducible disulfide groups. The resulting HBPs were characterised by Nuclear Magnetic Resonance Spectroscopy (NMR) and Gel Permeation Chromatography (GPC). Differential Scanning Calorimetry (DSC) was used to determine lower critical solution temperatures (LCSTs) of these copolymers, which are in the range of 17–57 °C. Moreover, the studies on the reducibility of HBPs and swelling behaviours of hydrogels synthesized from these HBPs were conducted. The results demonstrated that we have successfully synthesized hyperbranched polymers with desired dual responsive (thermal and reducible) and crosslinkable (via thiol-ene click chemistry) properties. In addition, these new HBPs carry the multiplicity of reactive functionalities, such as RAFT agent moieties and multivinyl functional groups, which can afford them with the capacity for further bioconjugation and structure modifications.

## 1. Introduction

Injectable hydrogels have advantages of homogeneously distributing cells and therapeutic molecules throughout the gels, and being able to fill cavities with irregular shapes and sizes. However, the challenge lies in finding suitable materials, which can solidify in situ to form 3D microenvironments with desired mechanical and biological properties. Linear or branched polymers with smart and crosslinkable properties, which change in response to external stimuli such as temperature, pH, and enzymes, have attracted much attention for such applications. Hyperbranched polymers (HPBs) have shown great advantages over their linear counterparts or even dendrimers due to their ease of synthesis, branched structures, and unique properties [1,2]. HPBs have high solubility, low solution viscosity, tailored mechanical strength, and a large number of easily accessible functional groups [2,3]. In addition, the ability of enclosing guest molecules and often uncommon self-assembly behaviour makes HBPs the preferred choice for many applications [4,5,6,7].

Multi-vinyl monomers (MVMs) are commonly used in the preparation of crosslinked materials. As was reported, polymerisation of MVMs often leads to insoluble crosslinked networks [8]. For years, only a low percentage of MVMs have been used in copolymerisations. In conventional free radical polymerisations (FRPs), the addition of a small amount of MVMs would lead to a crosslinked network when the conversion of monomer to polymer is less than 20% [9,10]. In recent years, MVMs have been successfully used in the syntheses of hyperbranched polymers via controlled radical polymerisation (CRP) methods, and soluble hyperbranched polymers with controlled molecular weights and degree of branching, tailored crosslinking density as well as well-defined 3D macromolecule structures were achieved which is impossible to achieve via conventional FRP [6,11,12].

CRP methods mainly include nitroxide mediated polymerisation (NMP), atom transfer radical polymerisation (ATRP), and reversible addition fragmentation chain transfer polymerisation (RAFT) [6,13]. RAFT polymerisation allows the control over the polymer synthesis by the simple addition of RAFT agent [7], and potentially dangerous or toxic metal catalysts are not required. In addition, RAFT process can better tolerate traces of impurities and is compatible with the broadest range of monomers and reaction conditions [14]. Moreover, an addition of RAFT agent in principle should not have any influence on polymerisation rate and radical concentration. The advantage of using CRP methods is that polymers with narrow polydispersity index (PDI) can be synthesized from readily available vinyl monomers. In recent years, more environmentally benign and industrially relevant alternative for synthesizing polymers with narrow PDI were also reported, such as low parts per million (ppm) techniques and microflow technology [15,16,17,18,19,20].

CRP methods have been used for the preparation of hyperbranched polymers through self-condensing vinyl polymerisation [21,22]. However, special inimers or transmers are required. We have previously succeeded in the synthesis of hyperbranched, photocrosslinkable, and thermoresponsive polymers using commercially available poly(ethylene glycol)methyl ether methacrylate (PEGMEMA), poly(propylene glycol)methacrylate (PPGMA), and ethylene glycol dimethacrylate (EGDMA) via atom transfer radical polymerisation (ATRP) and reversible addition-fragmentation chain transfer (RAFT) [23,24,25,26,27]. These PEGMEMA–PPGMA–EGDMA hyperbranched copolymers have been evaluated and demonstrated their suitability for use as injectable hydrogels. However, PEGMEMA–PPGMA–EGDMA polymers lack biodegradability under physiological conditions. The aim of this work was to afford biodegradability to the hyperbranched polymers by using disulfide diacrylate (DSDA) as the branching agent instead of EGDMA.

Zhao et al. synthesized highly branched degradable poly(dimethylamino-ethyl methacrylate) (PDMAEMA) by in situ de-ATRP using DSDA as the branching agent [28]. Degradation was observed with a faster reduction rate for hyperbranched structures in the presence of glutathione. The high degree of branching was achieved by using a high ratio of initiator/DMAEMA (1:8–1:32). Disulfide diacrylate is a symmetrical di-vinyl monomer. The di-vinyl functionality enables polymeric branching to occur across the methylidene groups. The divalent disulfide bond is susceptible to cleavage by polar reagents, both electrophiles and especially nucleophiles, to form thiol bonds [29]. It is known that disulfide bonds can be readily and selectively cleaved using various reducing reagents [30,31]. Disulfide-based branching agents are previously reported in the literature and were introduced to polymers with low solubility and high molecular weights (synthesized via ATRP). The addition of disulfide-based branching agents within the overall structure allowed the preparation of hydrogels that can undergo degradation (due to the presence of disulfide bonds) and produce soluble polymers at lower molecular weights [30,32,33,34].

**Scheme 1:**
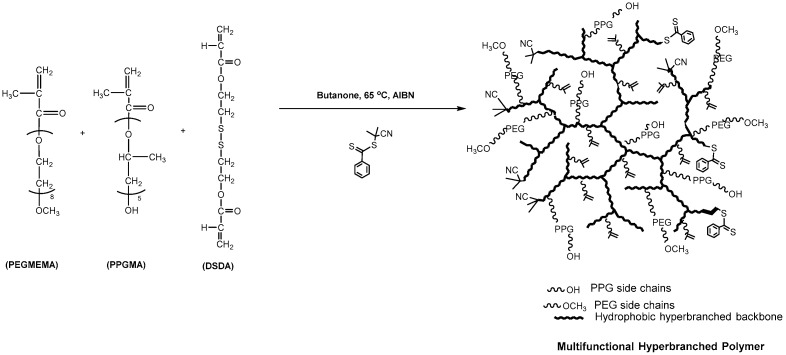
Synthesis of reducible thermoresponsive hyperbranched polymers with multivinyl functionality via reversible addition-fragmentation chain transfer (RAFT) copolymerisation of poly(ethylene glycol)methyl ether methacrylate (PEGMEMA), poly(propylene glycol)methacrylate (PPGMA), and disulfide diacrylate (DSDA).

In this work, new thermoresponsive HBPs were synthesized via one-pot RAFT copolymerisation of poly(ethylene glycol)methyl ether methacrylate (PEGMEMA), poly(propylene glycol)methacrylate (PPGMA), and disulfide diacrylate (DSDA) using 2-cyanoprop-2-yl dithiobenzoate as a RAFT agent (Scheme 1, Table 1). The copolymers were tailored to be cleavable under mild conditions via reduction and crosslinkable at body temperature via physical and chemical means. The resulted HBPs were characterised by NMR, GPC, and DSC, and the reducibility of the HBPs and swelling property of hydrogels prepared by these HBPs were studied.

## 2. Materials and Methods

### 2.1. Materials

Chemicals were purchased and used as received unless stated. The macromonomers employed in this work include poly(ethylene glycol)methyl ether methacrylate (PEGMEMA, *M*_n_ = 475 g/mol, Sigma-Aldrich, St. Louis, MO, USA) and poly(propylene glycol)methacrylate (PPGMA, *M*_n_ = 375 g/mol, Sigma-Aldrich). Bis(2-acryloyl)oxyethyl disulfide (DSDA—disulfide diacrylate), bis(thiobenzoyl) disulfide, and 2-cyanoprop-2-yl dithiobenzoate were synthesized and purified according to published methods [35,36,37] and characterised by ^1^H NMR. Azobisisobutyronitrile (AIBN, Sigma-Aldrich) was used as the initiator after being purified by re-crystallisation from methanol. The other chemicals used in this work include bis-2 hydroxyethyl disulfide (Sigma-Aldrich), acryloyl chloride (Sigma-Aldrich), triethylamine (Fisher Scientific, Hampton, NH, USA), sodium hydrogen carbonate (Fisher Scientific), sodium chloride (Fisher Scientific), anhydrous tetrahydrofuran (Sigma-Aldrich), dichloromethane (Sigma-Aldrich), sodium thiosulfate (anhydrous, Fisher Scientific), sodium sulfate (Fisher Scientific), 1N iodine aqueous solution (Sigma-Aldrich), pentaerythritol tetrakis(3-mercaptopropionate) (QT) (Sigma-Aldrich), dithiothreitol (DTT, Sigma-Aldrich), methylene chloride (Fisher Scientific), phenylmagnesium bromide (Sigma-Aldrich), carbon disulfide (Sigma-Aldrich), ethyl acetate (Fisher Scientific), diethyl ether (Fisher Scientific), hexane (Sigma-Aldrich), petroleum spirits 40–60 °C (Sigma-Aldrich), tetrahydrofuran (THF, Sigma-Aldrich), *n*-butanone (Sigma-Aldrich), deuterated chloroform (CDCl_3_, 99.8%, FluoroChem, Hadfield, UK), phosphate buffer powder (PBS, pH = 7.44, Sigma-Aldrich), solid iodine (Sigma-Aldrich), sodium hydride (60% in oil) (Sigma-Aldrich), and silica gel (60A, FluoroChem).

### 2.2. Methods

#### 2.2.1. Synthesis of Bis(thiobenzoyl) Disulfide, (2-Cyanoprop-2-yl Dithiobenzoate), and Disulfide Diacrylate (DSDA)

Bis(thiobenzoyl) disulfide, (2-cyanoprop-2-yl dithiobenzoate), and disulfide diacrylate (DSDA) were synthesized according to previously published methods, and the experimental details are provided in the Supplementary Information (Schemes S1–S3, Figure S1).

#### 2.2.2. Preparation of PEGMEMA–PPGMA–DSDA Copolymers via One-Pot RAFT Polymerisation

The conditions used for copolymerisation reactions were reported in Table 1. Here is a brief description of the typical reaction procedures used (Supplementary Information Scheme S4). Monomers PEGMEMA, PPGMA, and DSDA were prepared in 100 mL flask, and the molar ratio of [PEGMEMA]/[PPGMA]/[DSDA]/RAFT agent/[AIBN] is equal 20/70/10/1/0.2, i.e., PEGMEMA (1.80 g, 3.80 mmol), PPGMA (4.99 g, 13.30 mmol), DSDA (0.5 g, 1.90 mmol), RAFT agent 2-cyanoprop-2-yl dithiobenzoate (0.04 g, 0.19 mmol), and *n*-butanone (10 mL). The reaction flask was degassed for 10 min with argon, and AIBN (0.0328 g, 0.2 mmol) was added. The mixture was degassed for further 5 min. The solution was stirred at 500 rpm and then immersed in an oil bath at 65 °C and further stirred at 500 rpm. During the reaction, samples were withdrawn for GPC analysis (taken at required time points, filtered through a PVDF filter with 0.2 μm pore size, and then analysed). At the required reaction time (Table 1), polymerisations were terminated. Samples of the resultant polymers were purified by precipitation and then lyophilised. The purified polymers were subject to characterisations by GPC and ^1^H NMR.

#### 2.2.3. Characterisation of PEGMEMA–PPGMA–DSDA Copolymers

The resultant polymers were characterised by Gel Permeation Chromatography (GPC) and ^1^H NMR analysis. Number average molecular weight (*M*_n_), weight average molecular weight (*M*_w_) and polydispersity (*M*_w_/*M*_n_) were obtained by GPC (PL-50, Polymer Labs, Church Stretton, UK) with a Refractive Index (RI) detector. The GPC analyses were undertaken at 40 °C with a flow rate 1 mL/min while poly(methylmethacrylate) (PMMA) narrow standards were used. The columns used are PolarGel-M guard column (7.5 mm × 50 mm) with two main PLgel Mixed-C columns (7.5 mm × 300 mm) connected in series, with THF as an eluent. ^1^H NMR spectra were recorded on a 400 MHz Bruker Spectrometer (Bruker, Coventry, UK). MestReNovaLITE processing software was used to analyse NMR data. Samples were pre-dissolved in CDCl_3_ and the chemical shifts were referenced to tetramethylsilane (TMS). Lower Critical Solution Temperatures (LCSTs) were determined via two methods, i.e., visual observation and Differential Scanning Calorimetry (DSC) using the polymer concentration of 10 mg/mL in H_2_O. The values obtained from both methods are in good agreement (Table 1).

#### 2.2.4. Fabrication of Hydrogels by Thermal Gelation

Thermoresponsive PEGMEMA–PPGMA–DSDA copolymers (weight range between 100 and 500 mg) were dissolved in deionised water (1 mL) at 4 °C and incubated in an oven at 37 °C for 5 to 30 min. Gelation was defined as no flowing was observed within 10 s upon inversion of the vial by visual observation (see Supplementary Information Figure S2).

#### 2.2.5. Fabrication of Hydrogels by Michael Addition Reaction

Chemically crosslinked hydrogels from PEGMEMA–PPGMA–DSDA copolymers were synthesized using pentaerythritol tetrakis (3-mercaptopropionate), QT, as a thiol-functional crosslinker to react with acrylate functional groups in PEGMEMA–PPGMA–DSDA copolymers. Thiol functional groups in QT and the acrylate groups in the copolymer were used in an equal molar ratio. After gentle mixing the copolymers solutions of PEGMEMA–PPGMA–DSDA in PBS buffer (1 mL, pH 7.44) and QT by reversing the tubes up and down, the samples were left to incubate in an oven at 23 °C (room temperature) and 37 °C for 24 h (see Supplementary Information Figures S3 and S4). 

#### 2.2.6. Swelling Studies

Hydrogels of PEGMEMA–PPGMA–DSDA (weight range between 100 and 500 mg) were lyophilised, weighed individually, and immersed in phosphate buffer solution (2 mL, pH = 7.44) until equilibrium to test the maximum water uptake. The samples were weighed at regular time intervals, and swelling ratios were calculated out according to the weight changes of the gels [26]. Measurements were performed in triplicates.

#### 2.2.7. Reducibility Studies

The reducibility studies on the copolymers were conducted using DTT as the reducing agent to cleave the disulfide bonds. Two conditions were tested, i.e., one at a temperature above LCST and one at room temperature. Briefly, polymers (6 mg) were dissolved in THF (2 mL) and mixed with 1 M DTT solution (50 µL) and incubated in an oven at 50 °C or at room temperature for 5 h. Incubated samples were diluted with THF (2 mL) and analysed using GPC at different time points to monitor the progress of degradation. Similar reducibility tests on the copolymers were also conducted in water instead of in THF.

## 3. Results and Discussion

### 3.1. Synthesis and Characterisation of Reducible and Thermoresponsive Hyperbranched Polymers via One-Pot RAFT Polymerisation

The reaction conditions used for copolymerisations of PEGMEMA, PPGMA, and DSDA via RAFT were reported in Table 1. These RAFT polymerisations were carried out at 65 °C in butanone for the desired reaction time, while the reactions were monitored by GPC analysis. RAFT polymerisation is more versatile than ATRP approach because it does not require toxic metal catalysts and is applicable to a wider range of monomers [23].

**Figure 1:**
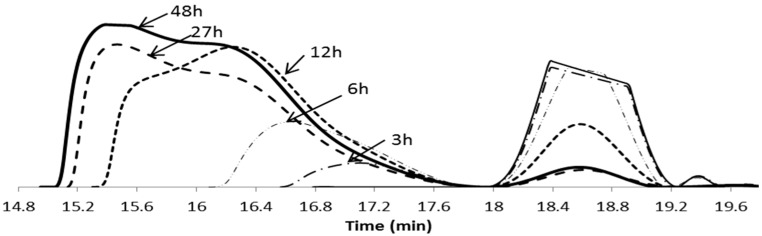
GPC traces from Refractive Index (RI) detector (entry 1 in Table 1) with the final polymer molecular weight of 12.7 kg/mol.

**Figure 2:**
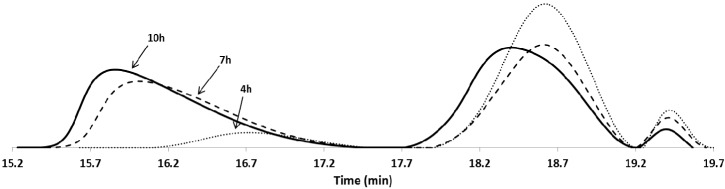
GPC traces from RI detector (entry 7 in Table 1) with the final polymer molecular weight of 10.2 kg/mol.

All reactions were aimed to achieve maximum monomer conversions while avoiding gelation. This is important for living polymerisations because the degree of polymerisation increases with monomer conversion. R4 and R6 had the same polymer composition and concentration, the difference was that polymer synthesis for R6 (entry 6) was terminated at a higher monomer conversion and thus had a higher molecular weight with slightly lower PDI than R4 (entry 4). In free radical copolymerisations of MVMs, resultant copolymers are often insoluble crosslinked structures at a very low monomer conversion. To date, polymerisation of MVMs is still difficult, thus special attention needs to be paid to the synthesis method and purification steps. While working on the synthesis of copolymers using MVMs via the RAFT method, monomer conversion was kept below the gelling point in order to prepare soluble hyperbranched copolymers. The molar ratio of initiator to vinyl monomer used in this study was 1:50 to 1:100, and high conversions were achieved without gelation. Moreover, the relatively low PDI values obtained (Table 1) demonstrated the nature of controlled chain growth of these polymerisation reactions. The reactions were monitored by GPC analysis, which showed the increase in molecular weight and polydispersity with monomer conversion (Figure 1 and Figure 2) and demonstrated that polymerisations progressed with narrower GPC traces at the beginning, and monomer peaks were decreasing with the reaction time. At the later stage of the reaction, the intermolecular coupling led to broader PDI values and hyperbranched structures formed. These data confirmed that the copolymer chains were growing over time. The process of generating hyperbranched polymers by using a divinyl cross-linker in the presence of a RAFT chain transfer agent should be regarded as a controlled radical polymerization process because it resulted in soluble HBPs with a relative narrow PDI at a high monomer conversion without gelation. The polymer chains grow over time in a linear fashion at low monomer conversion. However, the polymerisation kinetic does not follow the first order kinetics, in particular at the later stage of the polymerisation, due to the combination of polymer growing chain radicals.

**Figure 3:**
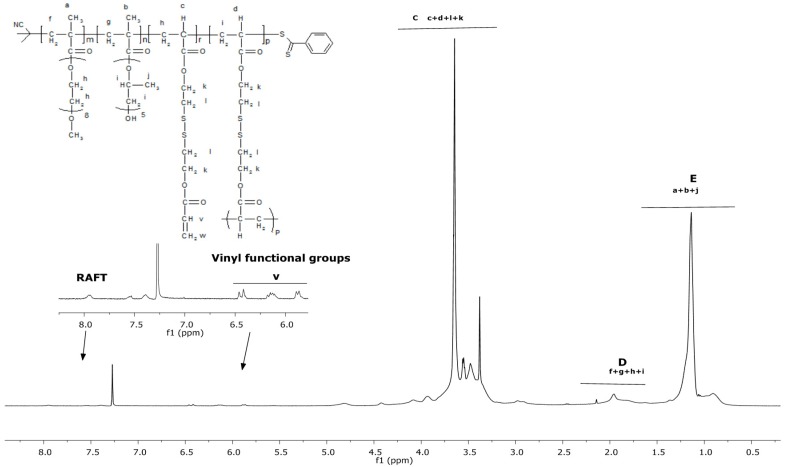
^1^H NMR of PEGMEMA–PPGMA–DSDA copolymer (R4, entry 4 in Table 1) prepared via RAFT polymerisation. Note: the spectrum shows clearly the double bonds within the structure and the copolymer composition (*m*, *n*, *r*, *p*) can be calculated from the integral data.

The hyperbranched structures of the copolymers were confirmed by ^1^H NMR. The chemical shifts were referenced to the lock CDCl_3_. The final composition of the copolymer is influenced by the reactivity of monomers and usually differs from the initial feed composition of the monomers. Polymer compositions (*m*, *n*, *r*, and *p* values in the macromolecule structure, Figure 3) were calculated using integration values (V, C, E, D) according to the peak assignments for ^1^H NMR analysis [25,26] (see Supplementary Information Equations (S1)–(S9)). The double bond content (DBC) and degree of branching (DOB) of the copolymers were calculated from the Equations (1) and (2). DBC represents the molar percentage of DSDA with free vinyl functional groups in the copolymer and the DOB represents the molar percentage of DSDA as branching units in the copolymer.

(1) $$\mathrm{Double}\;\mathrm{Bond}\;\mathrm{Content}\;\left(\mathrm{DBC}\right)\;\%=\frac{r}{\left(m+n+r+p\right)}\times 100$$

(2) $$\mathrm{Degree}\;\mathrm{of}\;\mathrm{Branching}\;\left(\mathrm{DOB}\right)\;\%=\frac{p}{\left(m+n+r+p\right)}\times 100$$

According to NMR analysis, the compositions of R4 and R7 are 24:49:27 and 22:45:33, respectively (Table 2). A higher degree of branching (up to 61.7 mol %) was achieved in polymer sample R7, while a low level of free vinyl groups was attained. However, this still allowed copolymers to react readily with thiol functional crosslinker (QT) via the Michael addition reaction to form a chemically crosslinked network. Reducing the amount of RAFT agent and initiator used in the reactions (comparing entry 7 with entry 4 in Table 1) led to polymers with higher DBC (from 0.5 to 1 mol %) and DOB (from 45.9 to 61.7 mol %).

### 3.2. Determination of Lower Critical Solution Temperatures (LCST) and the Formation of Physical Gels

By altering ratios of hydrophobic/hydrophilic fragments within the copolymer, molecular weight, and branching degree, the phase transition temperature of the polymer solution can be tailored. The more hydrophilic the copolymer is, the higher LCST is observed [27,38]. The LCSTs of the PEGMEMA–PPGMA–DSDA copolymers synthesized were tested by two methods, i.e., visual observation and DSC (Supplementary Information Figures S5 and S6). The lowest cloud point is usually referred to as the lowest critical solution temperature (LCST) [39]. The cloud point or LCST is concentration dependent, and it is commonly defined as the temperature at the first sign of turbidity by visual observation. The values obtained from both methods are in good agreement (Table 1). At the temperature above the LCST of each sample, the solution reversibly became cloudy, and gelation occurred when the polymer samples are at a sufficient concentration (greater than 20%). Gelation point was determined by visual observation as no flow upon inversion of the vial within 10 s. The solutions were clear and of a viscous nature below this temperature. By varying the monomer feed ratio in the copolymer synthesis, the LCSTs were tailored within the range of 17 to 57 °C (Table 1).

Due to thermal responsiveness, the copolymer solutions were found to be able to form physical gels at a concentration about 20% *w*/*v* (and above) when the temperature was raised beyond their LCSTs. It is known that the physical thermal gelation is reversible and also displays weak mechanical properties that might hold back their clinical applications [40,41,42]. Therefore, the chemical crosslinking can be introduced to enhance the mechanical properties of the gels [43].

### 3.3. Fabrication of Chemical Cross-Linked Hydrogels from Thiol-Ene Michael Addition Reaction Using PEGMEMA–PPGMA–DSDA Copolymers

Chemical crosslinking through click reactions of active functional groups such as thiols and acrylate under mild conditions has proven to be attractive for hydrogel synthesis as it can enhance mechanical properties of hydrogels and encapsulate bioactive ingredients, including cells [24,44,45]. Hydrogel fabrication via thiol-ene Michael addition reactions has offered versatility and improved workability, gelation time and in situ gelling at physiological conditions [46]. Moreover, this “click” reaction has limited equipment requirements, which is an attractive factor.

The Michael addition reaction between thiol and acrylate requires the presence of a base to act as a catalyst [47,48,49]. As the reaction is selective toward thiols under physiological conditions, the side reactions toward amines in the body are limited. Reaction precedes with the formation of a triethylammonium cation and a thiolate anion, a powerful nucleophile [47]. This type of reaction typically delivers gelation in a few minutes up to tens of minutes at physiological pH [50]. The basic conditions in this work were supplied by the use of PBS buffer (pH 7.44). Nucleophilic addition of thiol and diacrylate has been well studied in recent years [51]. Nucleophilic attack at the activated free vinyl groups of the polymer generates a strong base, able to deprotonate thiols and consequently results in the formation of thiolate anions, which can participate in the rapid formation of thiol-Michael addition products by the hydrothiolation of activated vinyl groups [52,53,54].

Reducible and thermoresponsive copolymers synthesised in this work can undergo the Michael addition reaction to form chemically crosslinked structures when a thiol-containing crosslinker is used due to the presence of free vinyl groups in the copolymers [55,56,57]. The vinyl groups in the copolymer structure were evidenced by ^1^H NMR spectrum with the three chemical shifts between 5.8 and 6.4 ppm (Figure 3). Michael addition reactions were performed using QT (pentaerythritol tetrakis, thiol functional crosslinker) at 23 and 37 °C respectively. It was recognized that potentially QT could also react with the crosslinking disulfides and dithiobenzoate end groups. However, on the hyperbranched polymers, there are multiple acrylate groups that more likely react with QT than disulfide or dithiobenzoate groups because of their higher reactivity. In addition, the low amount of QT was used (25% of free vinyl group), which could ensure QT preferably to react with acrylate groups, rather than disulfide and dithiobenzoate. In all cases, the gels were defined by visual examination as no flow when Eppendorf tubes were inverted (see Supplementary Materials Figures S2 and S3, respectively). The reactions at room temperature (23 °C) using 20 wt % and 40 wt % of purified copolymers resulted in milky solutions and a white precipitate (as seen in Figure S4, Supplementary Materials). These results indicated that the reaction occurred.

The low degree of free vinyl functional groups (about 0.5% molar ratio) in the prepared copolymers resulted in a soft chemically crosslinked hydrogel. The pinkish color of the froth indicated the presence of RAFT functional group in the hydrogel which could allow further modifications. Moreover, the amount of the free vinyl groups and branching degree could be further tailored by changing the molar ratio of PPGMA, DSDA, and PEGMEMA in the polymer synthesis. The increase of free vinyl groups can significantly improve the reactivity during the Michael addition and consequently change the crosslinking density of the hydrogel. Such gels may be optimised for tissue engineering applications that require different softness, pore sizes, and porosity.

### 3.4. Swelling

**Figure 4:**
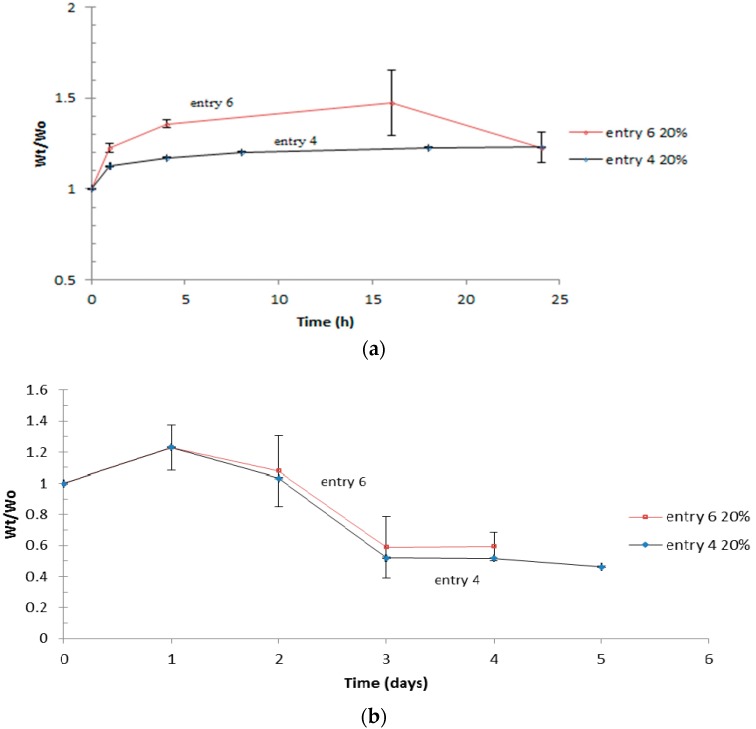
Swelling studies performed on chemically crosslinked 20% hydrogels prepared using R4 (entry 4, Table 1) and R6 (entry 6, Table 1) in PBS buffer (1 M, pH 7.4) at 37 °C. (**a**) in 24 h; (**b**) in 5 days.

The swelling of hydrogels prepared using PEGMEMA–PPGMA–DSDA copolymers was studied by soaking the gel samples in PBS buffer. Figure 4 shows the swelling curves for the 20% chemically crosslinked gels using R4 and R6 (entry 4 and entry 6 in Table 1) with QT at 37 °C.

The experimental results showed an increase in swelling for the hydrogels (prepared using the concentration of 20% copolymer) in PBS within 15 h (entry 6, R6) and 24 h (entry 4, R4), and the maximum swelling ratio was reached (Figure 4). After 24 h, it appeared that gel samples started to dissolve and in 4–5 days nearly half of the original weight was lost which could be caused by degradation of gels and the low chemical crosslinking due to a low percentage of free vinyl groups in the polymers.

**Figure 5:**
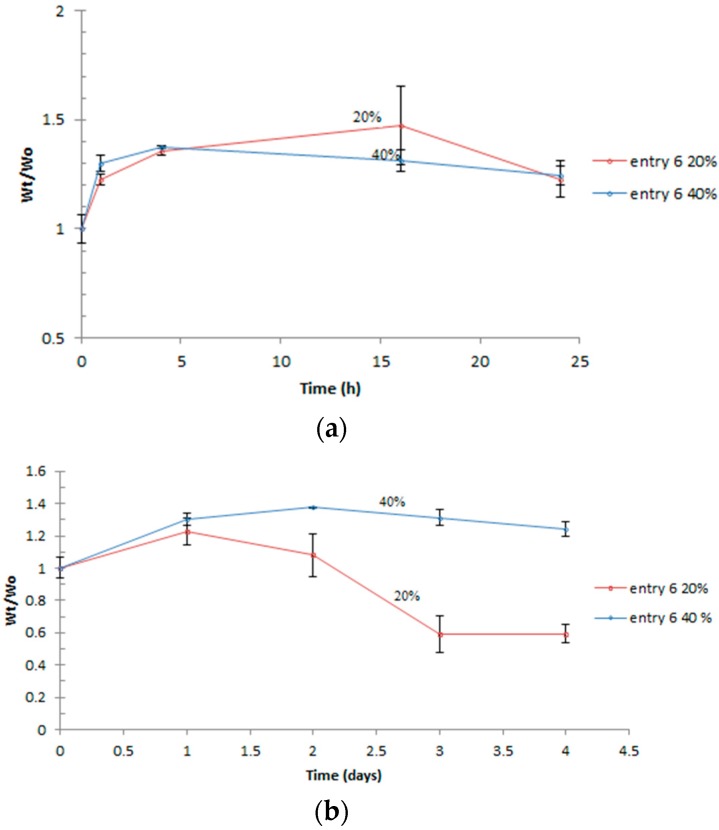
Swelling studies performed on chemically crosslinked 20% and 40% hydrogels using R6 (entry 6, Table 1) in PBS buffer (1 M, pH 7.4) at 37 °C after (**a**) 24 h and (**b**) 4 days.

Figure 5 shows the change in the swelling ratio of the hydrogels prepared at different concentrations (20% and 40%) using R6. For the hydrogels prepared using 20% PEGMEMA–PPGMA–DSDA, the swelling ratio reached a maximum of 1.48 after 16 h; in contrast, for 40% gel, it showed a similar maximum of swelling ratio of about 1.40 at 5 h. However, the error bars indicated the difference was not statistically significant, thus the swelling profiles were similar in the first 24 h for both hydrogels (i.e., 20% and 40%). Moreover, it demonstrated that the swelling and stability of the hydrogels is dependent on the polymer concentration used for preparing the hydrogels. A higher polymer concentration led to a more robust and mechanically stable hydrogel. The decrease in swelling ratio after three days could be due to degradation caused by breaking down of ester bond or disulfide bond facilitated by free thiol groups in the polymer structure. Also, some uncross-linked hyperbranched polymer chains could escape out of the network structure after prolonged swelling at a temperature above LCST.

### 3.5. Reducibility

The reducibility of thermoresponsive PEGMEMA–PPGMA–DSDA copolymers upon the addition of water-soluble dithiothreitol (DTT), a reducing agent, were studied using GPC. These degradation studies were performed in two different solvents, i.e., water and tetrahydrofuran (THF) (Supplementary Information Figures S7 and S8). Polymer samples tested were fully soluble in these solvents (at room temperature) as viscous solutions. The degradation test started using the copolymer sample, dissolved in 1 mL THF, then mixed with 1 M DTT to a final concentration of 0.1 M DTT, and incubated in an oven at 50 °C for 3 h.

**Figure 6:**
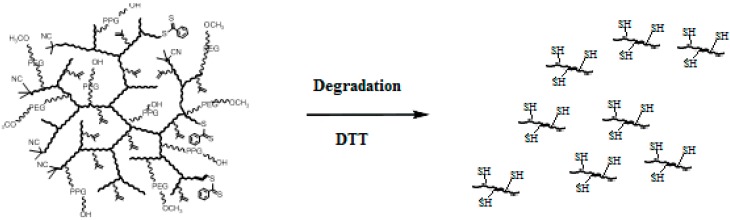
Schematic representation of the degradation PEGMEMA–PPGMA–DSDA polymer to primary chains by disulfide bond cleavage.

**Figure 7:**
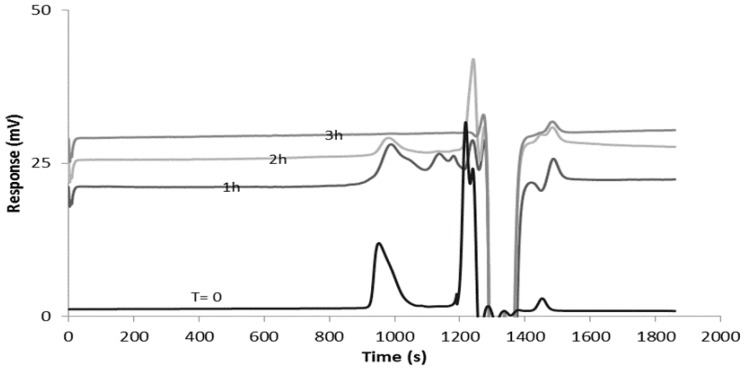
Overlaps of SEC traces recorded at various reaction time using the refractive index detector during the reductive degradation of the branched copolymer (PEGMEMA–PPGMA–DSDA/R:I, (20:70:10/1:0.2), entry 4, Table 1) with 0.1 M solution of DTT in THF at 20 °C.

Copolymers were quickly degraded into individual polymeric chains (Figure 6) and finally disappeared with time, as seen in Figure 7 (the GPC trace changed with reaction time, showing peaks with lower molecular weights, and finally no peak of the polymer was detected on the system); the chromatogram proved cleavage of the copolymer in the presence of DTT. The polymer peak on the GPC traces decreased with the time, and after 3 h there was no sign of polymer in the high molecular weight range. The disappearance of polymer peak indicates that this polymer in THF under conditions used was readily cleaved by DTT. ^1^H NMR performed on the samples after treatment with DTT confirmed the presence of low molecular weight polymer. The lack of detection of polymer chains after degradation using GPC could be due to the type columns employed in our GPC system, which do not work well for the polymers with low molecular weight. Using alternative columns suitable to detect oligomers could be the solution. In addition, polymer peaks disappearing could be caused by cross-linking of polymer chains due to the formation of disulfide bonds. However, it is also possible that microgelation may have occurred so that cleaved polymers were not detected by GPC.

Moreover, 0.1 M solution of DTT in water was added into the copolymer water solution to a final concentration of 0.001 M DTT. All samples were well mixed, then aliquoted to separate vials (incubated in a vacuum oven at 37 °C), and were analysed by GPC at required time points. Small tailing and an increase in PDI were recorded on GPC traces. This indicated that the reduction occurred at this mid-reducing conditions used.

## 4. Conclusions

New reducible and thermoresponsive PEGMEMA–PPGMA–DSDA hyperbranched copolymers with multiple acrylate groups and RAFT agent residues were successfully synthesised using one-pot RAFT copolymerisation. The molar feed ratios of monomers and reaction conditions were varied to tailor polymer properties (e.g., molecular weight and chemical composition) and to fine tune their LCSTs (between 17 and 57 °C). The polymers with a high degree of branching (up to 61.7 mol %) were achieved at high monomer conversion (up to 95%). No crosslinking or micro gelation was observed during the synthesis and purification processes. The copolymer solutions were found to form physical gels at the concentration about 20% *w*/*v* (and above) when the temperature was raised beyond their LCSTs. Chemical gelation was investigated using QT as the cross-linker via thiol-ene Michael addition reaction. Swelling and preliminary reducibility studies were also performed. Such dual responsive gels may be optimised for tissue engineering applications that require different softness, pore sizes, and porosity.

## Figures and Tables

**Table 1:** Synthesis of reducible and thermoresponsive polymers from RAFT copolymerisations of PEGMEMA, PPGMA, and DSDA at 65 °C in *n*-butanone.

Entry	Polymer samples obtained	*f* ^a^	RT ^b^ (h)	Cov ^c^ %	GPC RI			LCST °C	
					*M*_w_^d^	*M*_n_^e^	PDI ^f^	LCST ^g^	LCST ^h^
					kg/mol	kg/mol			
1	R1	70:20:10:5:1	48	95	12.7	9.0	1.40	-	-
2	R2	50:40:10:2:0.4	24	80	7.3	6.6	1.12	55	56
3	R3	50:40:10:1:0.2	24	64	11.3	9.8	1.15	57	57
4	R4	20:70:10:1:0.2	29	54	12.4	10.1	1.22	28	28
5	R5	15:65:20:1:0.2	25	58	11.2	8.9	1.26	22	23
6	R6	20:70:10:1:0.2	17	65	13.4	11.4	1.17	28	30
7	R7	20:70:10:0.5:0.1	11	46	10.2	8.7	1.16	18	17

^a^ Feed molar ratio of reactants: PEGMEMA:PPGMA:DSDA:RAFT agent: Initiator; ^b^ Reaction Time; ^c^ Percentage monomer conversion estimated by Gel Permeation Chromatography (GPC) using peak areas for monomer and polymer; ^d^ Weight-average molecular weight obtained by GPC; ^e^ Number-average molecular weight obtained by GPC; ^f^ Polydispersity obtained by GPC; ^g^ Lower Critical Solution Temperature (LCST) obtained by visual observation; ^h^ LCST obtained by Differential Scanning Calorimetry (DSC).

**Table 2:** Compositions of PEGMEMA–PPGMA–DSDA copolymers.

Polymer samples	*f* ^a^ PEGMEMA:PPGMA:DSDA	*f* ^b^ PEGMEMA:PPGMA:DSDA	Double bond (mol %)	Branching degree (mol %)
R4	20:70:10	24:49:27	0.5	45.9
R7	20:70:10	22:45:33	1	61.7

^a^ Feed molar ratio of monomer and comonomers; ^b^ Polymer composition.

## Supplementary Materials

The following are available online at www.mdpi.com/2073-4360/9/9/443/s1, Scheme S1: Synthesis of bis(thiobenzoyl) disulfide, Scheme S2: Synthesis of 2-cyanoprop-2-yl dithiobenzoate (CPDB), Scheme S3: Synthesis of bis(2-acryloyl)oxyethyl disulfide (DSDA), Scheme S4: Synthesis of thermoresponsive hyperbranched polymers with multivinyl functionality via conventional RAFT polymerisation using RAFT agent 2-cyanoprop-2-yl dithiobenzoate, Figure S1: ^1^H NMR of bis(2-acryloyl)oxyethyl disulfide (DSDA), Figure S2: Thermally induced gelation from 20% copolymer solution (PEGMEMA–PPGMA–DSDA/R:I (20:70:10/1:0.2), entry 4 in Table 1), Figure S3: 20 wt % polymer solution in PBS buffer (PEGMEMA–PPGMA–DSDA/R:I (20:70:10/1:0.2), entry 4 in Table 1) undergo Michael addition-type reaction: (a) 1 min after mixing with QT (1:1 vinyl group to SH); (b) 0.5 h after mixing with QT (1:1 vinyl group to SH) and incubated at 37 °C, Figure S4: 20 wt % polymer solution in PBS buffer (PEGMEMA–PPGMA–DSDA/R:I (20:70:10/1:0.2), entry 4 in Table 1), Michael addition-type reaction at room temperature (24 h after mixing with QT, 1:1 vinyl group to SH), white precipitate present in Eppendorf tube, Figure S5: LCST–DSC measurement for thermoresponsive )PEGMEMA–PPGMA–DSDA/R:I (20:70:10/1:0.2); entry 4, Table 1), Figure S6: LCST–DSC measurement for thermoresponsive (PEGMEMA–PPGMA–DSDA/R:I (50:40:10/2:0.4); entry 2, Table 1), Figure S7: GPC traces recorded at different time points (a) 0 h and (b) 5 h during the reductive degradation of (PEGMEMA–PPGMA–DSDA/R:I (20:70:10/1:0.2), entry 4 in Table 1) using 0.1 M solution of DTT in THF at 50 °C, Figure S8: GPC traces recorded at different time points (a) 0 h and (b) 5 h during the reductive degradation of (PEGMEMA–PPGMA–DSDA/R:I (20:70:10/1:0.2), entry 4 in Table 1) using 0.001 M solution of DTT in water at 50 °C.
