# Supplementary material for: Thermoresponsive and Reducible Hyperbranched Polymers Synthesized by RAFT Polymerisation
Source: Polymers (Basel). 2017 Sep 13;9(9):443. doi: 10.3390/polym9090443 (PMC6418797; doi:10.3390/polym9090443)
Supplement: Supplementary file 1 [file polymers-09-00443-s001.pdf]

polymers

Supplementary Information (SI)

Thermoresponsive and Reducible Hyperbranched Polymers Synthesized by RAFT Polymerisation

Anna Tochwin [1], Alaa El-Betany [1], Hongyun Tai [1,*], Kai Yu Chan [1], Chester Blackburn [1] and Wenxin Wang [2]

[1] School of Chemistry, Bangor University, Deiniol Road, Bangor, Gwynedd LL57 2UW, UK; annatoffi.at@gmail.com (A.T.); alaaelbetany@gmail.com (A.El-B.); chu037@bangor.ac.uk (K.Y.C.); chu230@bangor.ac.uk (C.B.)
[2] Charles Institute of Dermatology, University College Dublin, Dublin 4, Ireland; wenxin.wang@ucd.ie
* Correspondence: h.tai@bangor.ac.uk (H.T.); Tel.: +44-(0)1248-382-383; Fax: +44-(0)1248-370-528

Experimental

Synthesis of Bis(thiobenzoyl) Disulfide

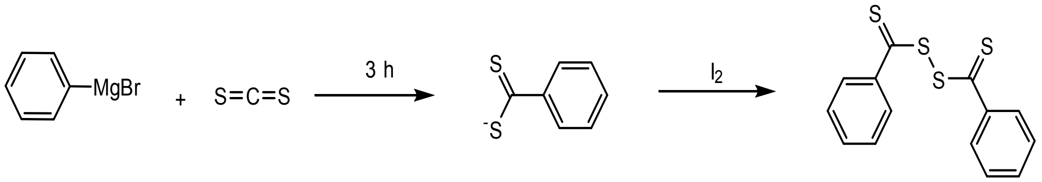

Scheme S1. Synthesis of bis(thiobenzoyl) disulfide

The compound was prepared by placing 1M solution of phenylmagnesium bromide (100 mL, 1M in THF) in a two-neck round-bottom flask equipped with a condenser, magnetic stirrer and nitrogen atmosphere. The flask was cooled in ice bath to 0 °C and carbon disulfide (8.36 mL, 139 mmol) was added dropwise over 20 min. The solution was stirred for 1h at 0 °C and another 2.5 h at room temperature. The solvent was then removed under vacuum and the resulting deep red viscous liquid was dissolved in a diluted K_2CO_3 solution (8 g in 200 mL), filtered and washed with diethyl ether (2 × 100 mL). The aqueous phase was collected and poured into a round-bottom flask equipped with magnetic stirrer. An aqueous solution of iodine 1.0 N (90 mL, 100 mmol) was then added dropwise over 30 min. During the addition, the solution started to change colour from dark red/purple to pink as the disulfide precipitated. After elimination of excess of I_2 with a few crystals of $Na_2S_2O_3$, the mixture was extracted with methylene chloride, dried over sodium sulfate, evaporated and dried in vacuum oven at 40 °C. A pink/light red powder was obtained (70.7%) and characterised by ^{1}H NMR. The crude product was used for the subsequent reaction without further purification.

^{1}H NMR (400 MHz, $CDCl_3$), δ(PPM): 7.48 (m, m-ArH, 4H); 7.64 (m, p-ArH, 2H); 8.11 (m, o-ArH, 4H).

Preparation of (2-cyanoprop-2-yl dithiobenzoate)

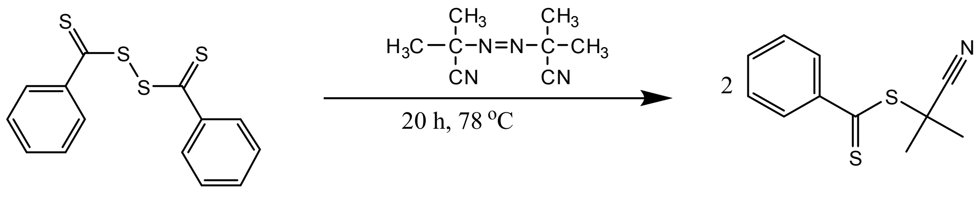

Scheme S2. Synthesis of 2-cyanoprop-2-yl dithiobenzoate (CPDB).

A solution of bis(thiobenzoyl) disulfide (0.858 g, 2.8 mmol) and the azo compound (AIBN) (0.532 g, 3.24 mmol) in ethyl acetate (100 mL) was degassed in a 250 mL round-bottom flask equipped with condenser and magnetic stirring and then refluxed under nitrogen atmosphere for 20 hrs. The solvent was evaporated and the product purified by flash column chromatography (eluent:hexane/DCM 3:2). A red-purple oily liquid (0.84 g, 68%) was obtained. The product was characterised by ^{1}H-NMR.

^{1}H NMR (400 MHz, $CDCl_3$) δ(PPM): 1.97 (s, 6H, CH3), 7.41 (m, 2H, ArH), 7.58 (m, 1H, ArH), 7.94 (m, 2H, ArH).

Synthesis of Disulfide Diacrylate (DSDA)–Bis(2-acryloyl)oxyethyl disulfide

Disulfide diacrylate (DSDA) was prepared using acryloyl chloride and bis-2 hydroxyethyl disulfide in anhydrous THF in the presence of triethylamine (Scheme S3). After the reaction, the reaction mixture was filtered to remove the triethylamine hydrochloride. The crude product was treated with aqueous basic salts to remove any remaining by-products or un-reactants, then was further purified by column chromatography to give an extra pure DSDA product which was analysed by ^{1}H NMR (Figure S1). A typical procedure for this synthesis is described below.

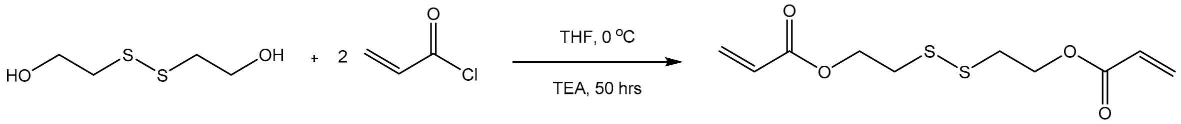

Scheme S3. Synthesis of bis(2-acryloyl)oxyethyl disulfide (DSDA) - disulfide diacrylate.

Bis-2 hydroxyethyl disulfide (7.70 g, 50 mmol) and triethylamine (40.5 g, 400 mmol) were weighed into a round-bottom flask, equipped with a magnetic stirrer bar. Anhydrous THF (150 mL) was added into the flask, immersed in an ice bath and purged with nitrogen for 20 min, at 0 °C. Acryloyl chloride (16.3 mL, 200 mmol) was then added dropwise to the reaction mixture, and the heterogeneous solution was left to stir for 50 h. The reaction mixture was filtered to remove the triethylamine hydrochloride by-product. The solvent was removed by rotary evaporation, and the crude product (brownish viscous oil) was dissolved in chloroform. Purification required washing organic phase with deionised water (3 × 300 mL), sodium hydrogen carbonate (3 × 300 mL) and brine (3 × 300 mL). The organic layer was stirred with anhydrous magnesium sulphate for 28 h. The crude product (9.82 g, 75%) was filtered to remove the magnesium sulphate, and was purified by column chromatography using silica gel as the stationary phase and dichloromethane as the eluent. The final disulfide diacrylate (DSDA) product was obtained as pale viscous yellow oil (4.80 g, 37%) and stored in the freezer, under nitrogen, in the absence of light prior to use. The purified DSDA was characterised by ^{1}H NMR.

^{1}H NMR (400 MHz, $CDCl_3$), δ(PPM): 2.98 (t, 2H); 4.43 (t, 2H); 5.86 (d, 1H); 6.13 (dd, 1H); 6.44 (d, 1H).

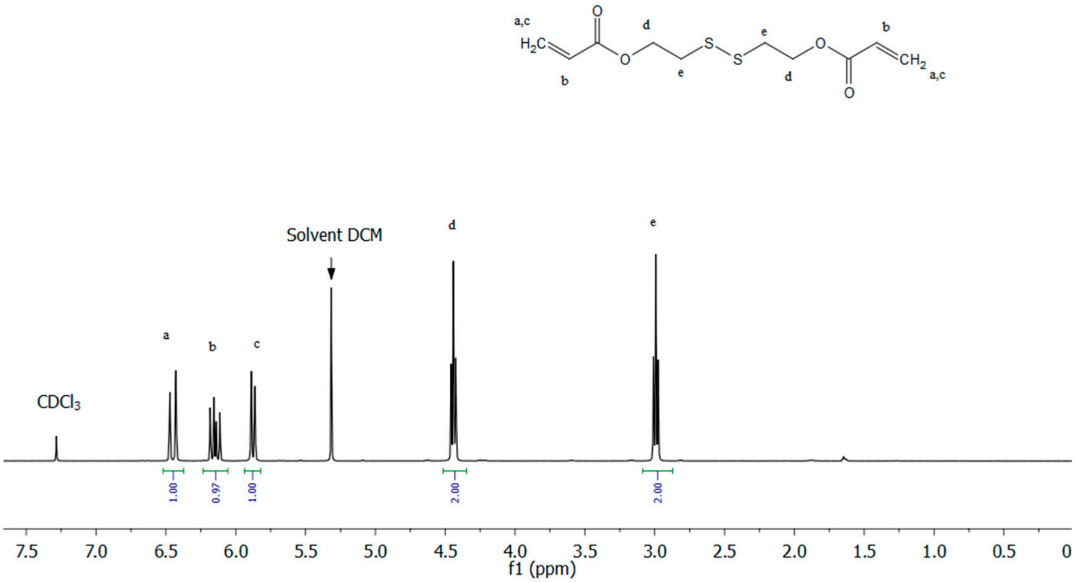

Figure S1. ^{1}H NMR of bis(2-acryloyl)oxyethyl disulfide (DSDA).

Preparation of PEGMEMA–PPGMA–DSDA Copolymers via one-pot RAFT Polymerisation

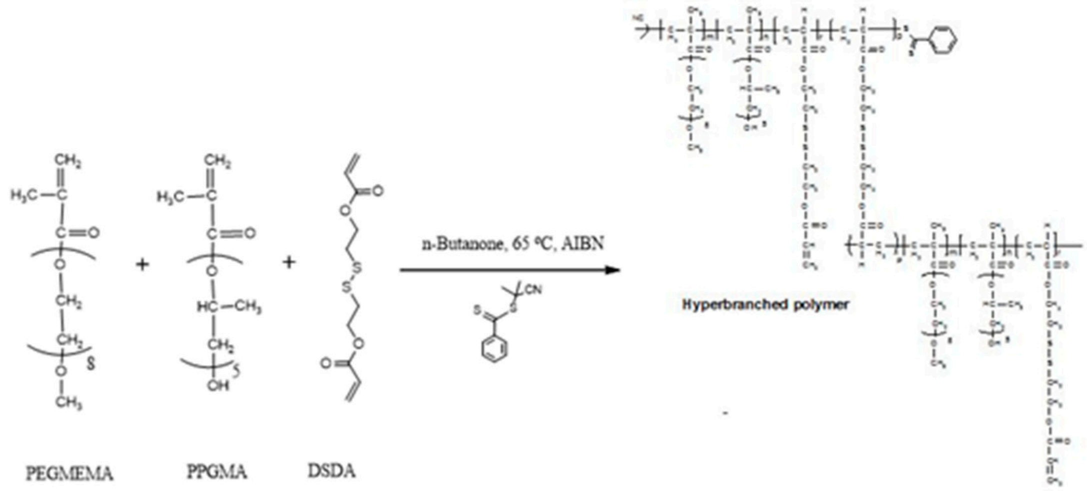

Scheme S4. Synthesis of thermoresponsive hyperbranched polymers with multivinyl functionality via conventional RAFT polymerisation using 2-cyanoprop-2-yl dithiobenzoate as the RAFT agent.

Composition of the Copolymer Calculated According to the Integration by ^{1}H NMR

The composition of the copolymer, represented by *m*, *r*, *n* and *p* values in the macromolecule structure (in Figure 3), was calculated from the integral data C, D, E and V respectively, according to equations s5–s8. These equations were derived from the four equations s1–s4 established for the proton integral regions of C, D, E, and V, respectively.

So, polymer compositions (*m*, *n*, *r*, and *p*) were calculated using equations s5 to s8. The characteristic peaks at chemical shifts of 6.1 and 5.6 ppm are attributed to the vinyl functional groups in the copolymer and the others are assigned as indicated in Figure 3.

Equations (s1 to s9) outline the calculations:

$$3r = V \tag{1}$$

$$35m + 15n + 9r + 9p = C \tag{2}$$

$$2m + 2n + 2r + 2p = D \quad (3)$$

$$3m + 18n = E \quad (4)$$

$$r = V/3 \quad (5)$$

$$n = 0.058E - 0.013C + 0.03B \quad (6)$$

$$m = 0.08C - 0.18D - 0.013E \quad (7)$$

$$p = 0.65D - 0.044E - 0.067C - 0.33V \quad (8)$$

$$r + m + n + p = 100 \quad (9)$$

Where: m = PEGMEMA; n = PPGMA; r = DSDA; p = Hyperbranched DSDA.

Double bond content and branching degree were calculated according to Equation 1 and Equation 2 in main manuscript.

Fabrication of Hydrogels by Thermal Gelation

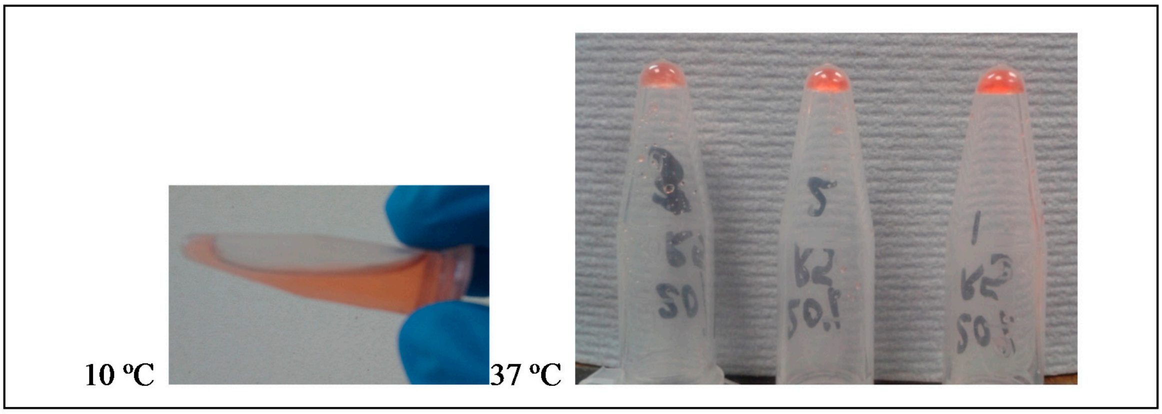

Figure S2. Thermally induced gelation from 20% copolymer solution (PEGMEMA–PPGMA–DSDA/R:I (20:70:10/1:0.2), entry 4 in Table 1).

Fabrication of Hydrogels by Michael Addition Reaction

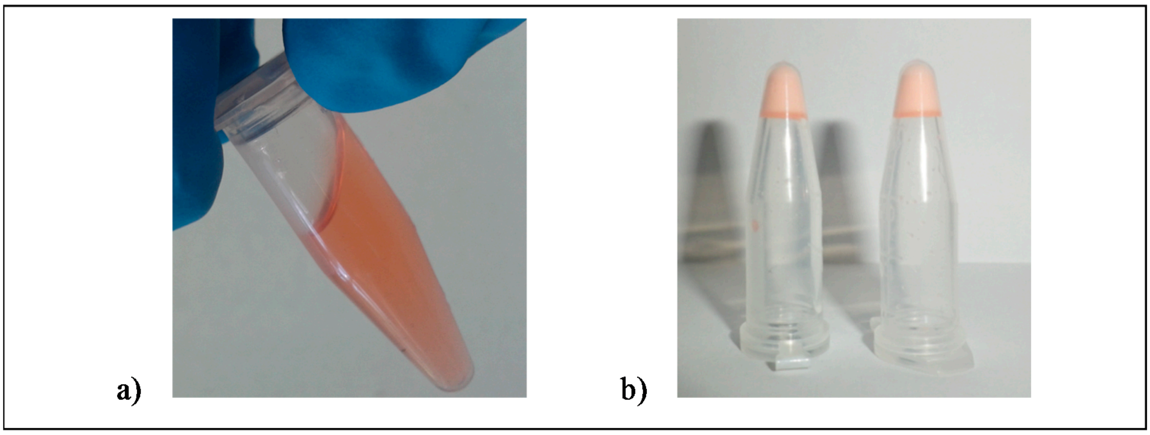

Figure S3. 20 wt % polymer solution in PBS buffer (PEGMEMA-PPGMA-DSDA/R:I (20:70:10/1:0.2), entry 4 in Table 1) undergo Michael addition-type reaction: a) 1 min after mixing with QT (1:1 vinyl group to SH); b) 0.5 h after mixing with QT (1:1 vinyl group to SH) and incubated at 37 °C.

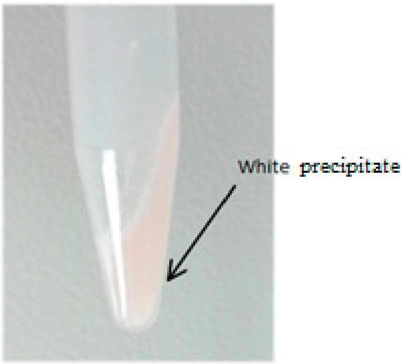

Figure S4. 20 wt % polymer solution in PBS buffer (PEGMEMA–PPGMA–DSDA/R:I (20:70:10/1:0.2), entry 4 in Table 1), Michael addition-type reaction at room temperature (24 h after mixing with QT, 1:1 vinyl group to SH), white precipitate present in Eppendorf tube.

Lower Critical Solution Temperatures (LCSTs) Determined via Differential Scanning Calorimetry (DSC) (Representative Data of DSC-thermograms)

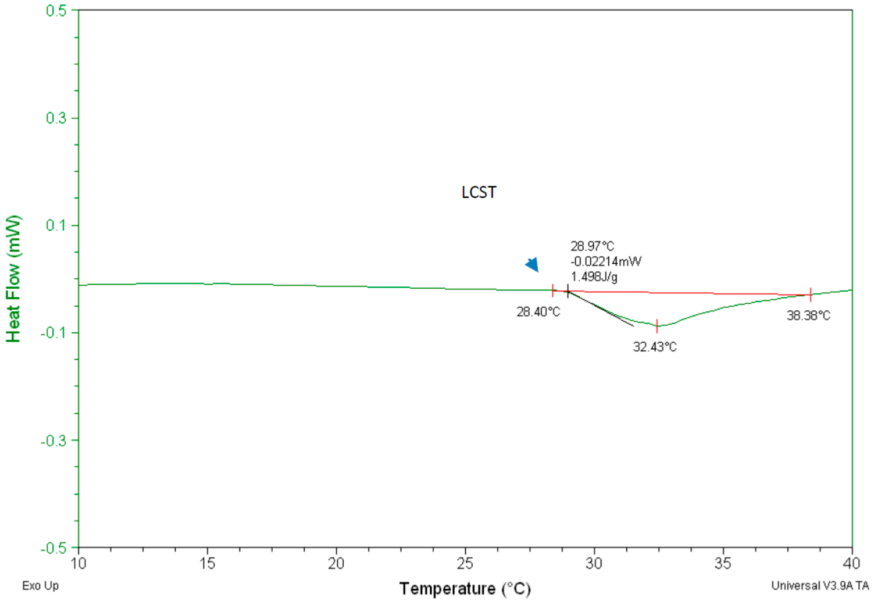

Figure S5. LCST–DSC measurement for thermoresponsive PEGMEMA–PPGMA–DSDA/R:I (20:70:10/1:0.2);entry 4, Table 1.

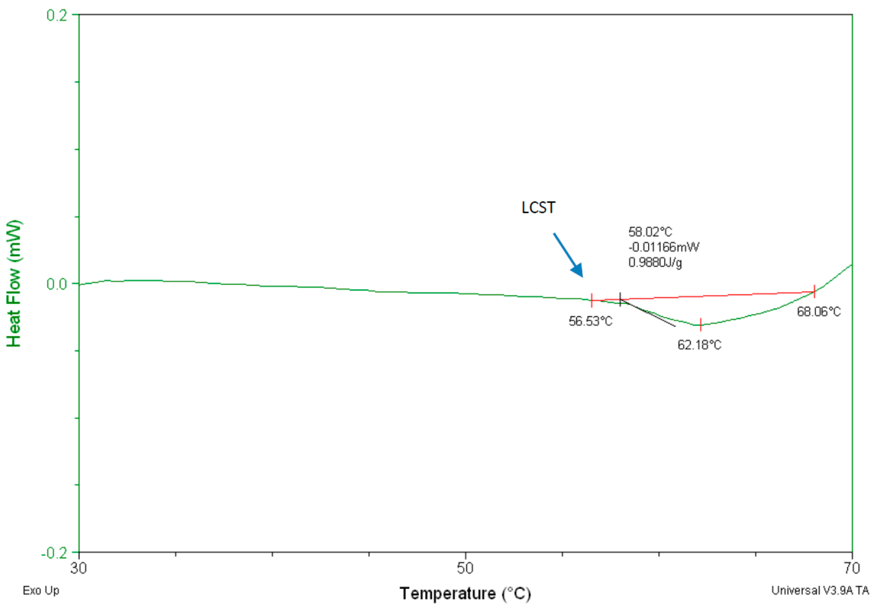

Figure S6. LCST–DSC measurement for thermoresponsive PEGMEMA–PPGMA–DSDA/R:I (50:40:10/2:0.4); entry 2, Table 1.

Degradation Studies

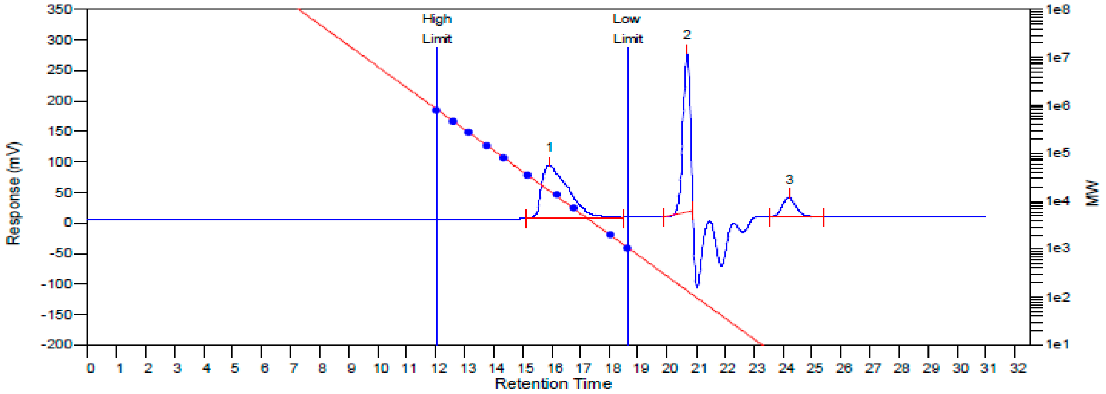

(a)

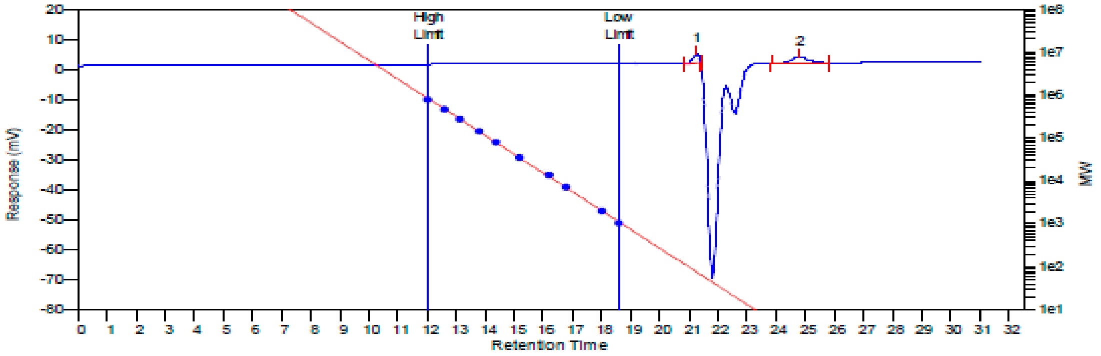

(b)

Figure S7. GPC traces recorded at different time points a) 0 h and b) 5 h during the reductive degradation of PEGMEMA–PPGMA–DSDA/R:I (20:70:10/1:0.2), entry 4 in Table 1) using 0.1M solution of DTT in THF at 50 °C.

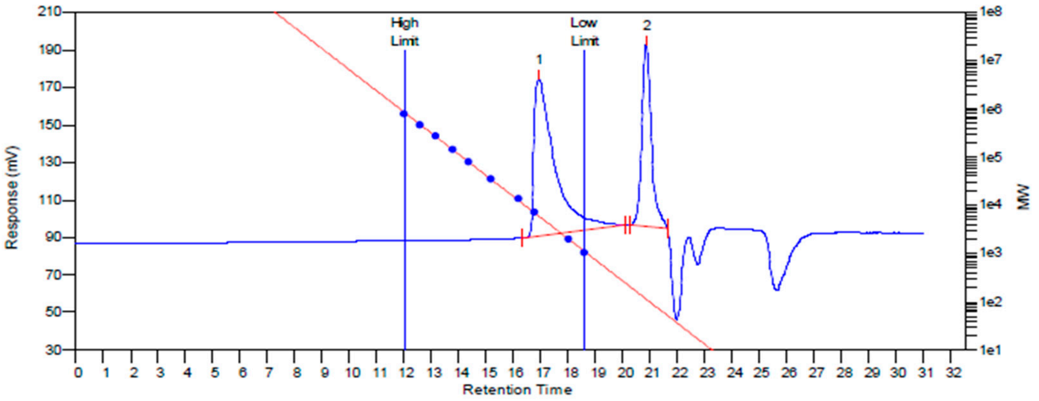

MW Averages

Peak No	Mp	Mn	Mw	Mz	Mz+1	Mv	PD
1	5948	3004	4334	5097	5559	4195	1.44274
2	114	107	111	115	118	110	1.03738

(a)

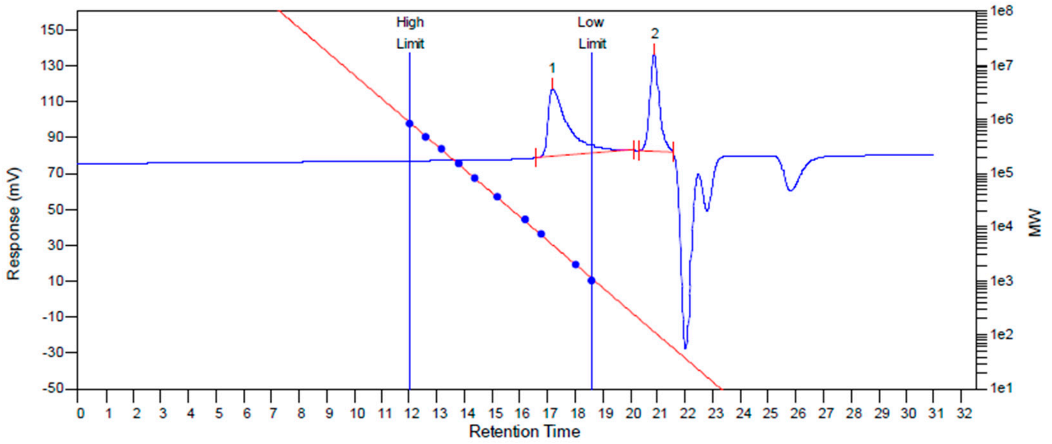

MW Averages

Peak No	Mp	Mn	Mw	Mz	Mz+1	Mv	PD
1	4623	2380	3412	4034	4416	3300	1.43361
2	114	108	111	115	118	111	1.02778

(b)

Figure S8. GPC traces recorded at different time points a) 0 h and b) 5 h during the reductive degradation of PEGMEMA-PPGMA-DSDA/R:I (50:40:10:2:0.4), entry 2 in Table 1) using 0.001M solution of DTT in water at 50 °C.

References

1. Ting, S. R. S.; Min, E. H.; Zetterlund, P. B.; Stenzel, M. H. Controlled/Living ab Initio Emulsion Polymerization via a Glucose RAFT stab : Degradable Cross-Linked Glyco-Particles for Concanavalin A/ Fim H Conjugations to Cluster E. coli Bacteria. *Macromolecules* **2010**, *43*, 5211–5221.
2. Benaglia, M.; Rizzardo, E.; Alberti, A.; Guerra, M. Searching for More Effective Agents and Conditions for the RAFT Polymerization of MMA: Influence of Dithioester Substituents, Solvent, and Temperature. *Macromolecules* **2005**, *38*, 3129–3140.

3. Milovanovic, M. B.; Avaramovic, M.; Katsiksa, L.; Popovic, I. G. Simplification of the synthesis of the reversible addition–fragmentation chain transfer agent 2-(2-cyanopropyl)-dithiobenzoate. *J. Serbian Chem. Soc.* **2010**, *75*, 1711–1719.

© 2017 by the authors. Submitted for possible open access publication under the terms and conditions of the Creative Commons Attribution (CC BY) license (http://creativecommons.org/licenses/by/4.0/).
